# The Potential Role of Neurophysiology in the Management of Multiple Sclerosis-Related Fatigue

**DOI:** 10.3389/fneur.2020.00251

**Published:** 2020-04-22

**Authors:** Fioravante Capone, Francesco Motolese, Emma Falato, Mariagrazia Rossi, Vincenzo Di Lazzaro

**Affiliations:** ^1^Unit of Neurology, Neurophysiology, Department of Medicine, Università Campus Bio-Medico di Roma, Rome, Italy; ^2^NeXT: Neurophysiology and Neuroengineering of Human-Technology Interaction Research Unit, Campus Bio-Medico University, Rome, Italy

**Keywords:** multiple sclerosis, fatigue, neurophysiology, non-invasive brain stimulation, TMS, tDCS

## Abstract

Fatigue is a very common symptom among people with multiple sclerosis (MS), but its management in clinical practice is limited by the lack of clear evidence about the pathogenic mechanisms, objective tools for diagnosis, and effective pharmacological treatments. In this scenario, neurophysiology could play a decisive role, thanks to its ability to provide objective measures and to explore the peripheral and the central structures of the nervous system. We hereby review and discuss current evidence about the potential role of neurophysiology in the management of MS-related fatigue. In the first part, we describe the use of neurophysiological techniques for exploring the pathogenic mechanisms of fatigue. In the second part, we review the potential application of neurophysiology for monitoring the response to pharmacological therapies. Finally, we show data about the therapeutic implications of neurophysiological techniques based on non-invasive brain stimulation.

## Introduction

Fatigue is a very common symptom in multiple sclerosis (MS) and produces significant detrimental effects on the quality of life (1). Despite its prevalence and impact, the management of fatigue in clinical practice is often challenging since the underlying pathophysiological mechanisms have not been well-elucidated (2), pharmacological treatments have limited efficacy (3), and fatigue assessment is commonly based exclusively on self-report questionnaires (4).

Although the advent of magnetic resonance imaging (MRI) significantly changed the overall management of MS, the role of neurophysiology remains of great importance in the functional evaluation of specific pathways such as visual, somatosensory, auditory, and motor systems and in the study of the central and the peripheral mechanisms of sensorimotor integration. Fatigue is a complex symptom including motor, cognitive, and psychological aspects, but through neurophysiological techniques, it is possible to evaluate mainly motor fatigue, from both research and clinical perspectives. Motor fatigue can be classified as central or peripheral. By definition, peripheral fatigue is the inability to generate force at the muscle level, while central fatigue refers to changes arising from the neural networks in the brain and the spinal cord, causing a lack of drive to the muscles.

The alterations occurring at the neuromuscular level cannot fully explain the phenomenon of fatigue (5), and in the last few years, different studies have speculated over the meaning and magnitude of the contribution of the central nervous system (CNS). In particular, in MS, fatigue seems to arise from the disruption of a complex neural network involving the cerebral cortex, the thalamus, and the basal ganglia (6–8). Similarly also in other neurological conditions such as Parkinson's disease and stroke, different supraspinal structures are considered to be key players in fatigue generation (9).

In this scenario, neurophysiological techniques can play a decisive role in the assessment of the pathophysiology of MS-related fatigue, thanks to their ability to provide objective measures and to explore the peripheral and the central structures of the nervous system, with excellent time resolution. Besides that, various studies have also demonstrated good correlations between neurophysiological parameters and disability measures (10), highlighting the usefulness of neurophysiology in monitoring disease evolution and response to therapy.

Finally, several studies have evaluated the therapeutic implications of neurophysiological techniques based on non-invasive brain stimulation (NIBS) in different neuropsychiatric diseases such as stroke, depression, dementia, and movement disorders (11–13). In particular, in MS, promising results have been obtained in the treatment of disabling symptoms such as spasticity (14) and fatigue (15).

In this review, we will provide an outline of the current evidence about the potential role of neurophysiology in the management of MS-related fatigue. In the first part, we will describe the potential application of neurophysiological techniques for exploring the pathogenic mechanisms of fatigue. Then, we will report on the potential use of neurophysiology for measuring fatigue and monitoring the response to symptomatic therapies. In the third part, we will review the potential application of neuromodulation as an innovative treatment for fatigue. Eventually, we will discuss the limitations and the shortcomings of available data, highlighting the key challenges in the field and suggesting some directions for future research.

## Neurophysiology as Investigating Tool for The Pathogenic Mechanisms of Fatigue

During a physical effort, there is a progressive decline of firing rate of spinal motoneurons (16), but the significance of such phenomenon is not clear as it can be interpreted as exhaustion or as fatigue adaptation.

Most studies reported that MS patients present lower strength values of maximal voluntary contraction (MVC) in comparison to healthy subjects (17–20), and the decrease of these values is positively correlated with fatigue perception (21). The fall of muscle force (and MVC as well) could be related to a submaximal voluntary drive, which is known as central activation failure (CAF) (9). CAF can be evaluated by the twitch-interpolated technique, in which the subjects are asked to perform a MVC in a given muscle and an electrical stimulus is subsequently applied to the motor nerve supplying the tested muscle. If there is a further increase of muscle force after electrical stimulation, then the muscle's voluntary central drive was not at its maximum, thus demonstrating CAF. Using this technique, Steens et al. (22) showed a decrease of voluntary activation during fatiguing exercise in people with MS (PwMS) in comparison to healthy subjects, probably due to insufficient CNS compensatory mechanisms. The reduction of voluntary activation seems to be particularly important in the pathogenesis of fatigue in patients with secondary-progressive MS as compared to relapsing–remitting MS (23).

Electromyography (EMG) allows quantifying the reduction of amplitude or frequency of muscle action potentials (MAP) during a fatiguing task. Surface EMG (sEMG) is a non-invasive technique in which electrodes placed on the skin record electrical muscle activity (24, 25). In particular, the amplitude of the sEMG signal is considered as a measure of voluntary drive to peripheral structures (9). Muscle contraction is characterized by the progressive recruitment of different motor units, depending on their size, biochemical features, and fatigability (26, 27). The development of muscular fatigue produces specific changes in EMG signal, consisting in an initial increase and then in the decrease of MAP amplitude (28, 29), a reduction of median frequency of discharge, and a reduction of motor conduction velocity along fatigued muscle fibers (28, 30, 31).

These phenomena, also present in healthy subjects, are more evident in PwMS. For instance, Eken et al. found that prolonged walking produces a significant decrease of EMG median frequency with a corresponding increase of the root mean square of the EMG signal of the soleus muscle (32). Similar changes of EMG parameters have also been found in the upper limb by Severijns et al. (33) in a cohort of PwMS after a protocol of repetitive shoulder anteflexion movements. Interestingly, these changes in EMG parameters are present even without a clear performance decline and are not directly correlated with the level of perceived fatigue. These findings suggest that peripheral mechanisms cannot fully explain the development of fatigue and that central mechanisms could also be involved. In this regard, different neurophysiological methods can be used to study the contribution of CNS.

Electroencephalography (EEG) allows evaluating the role of cortico-cortical connections. Using this technique, Leocani et al. (34) investigated the correlation between fatigue severity [measured through the Fatigue Severity Scale (FSS) questionnaire] and EEG parameters consisting of event-related desynchronization (ERD) and event-related synchronization (ERS). They found that, in PwMS compared to healthy controls, FSS correlated positively with ERD over midline frontal structures during movement and inversely with contralateral sensorimotor ERS after movement. These findings suggest an overactivation of the frontal regions in fatigued patients, a possible expression of a compensatory mechanism for the subcortical dysfunction causing fatigue.

Transcranial magnetic stimulation (TMS) is a non-invasive brain stimulation technique that can be used to explore the contribution of the different structures of the CNS to fatigue generation. Indeed single-pulse TMS allows evaluating the functionality of the corticospinal tract by recording the amplitude and the latency of motor-evoked potentials (MEP), while paired-pulse TMS provides insight into the cortico-cortical connections. Moreover, repetitive TMS (rTMS) protocols are known to induce short- and long-term modifications of cortical excitability, thus reflecting plasticity changes at the cortical level.

In healthy subjects, MEP amplitude increases during a fatiguing exercise and reduces after its end (35). In MS patients, results are more variable because some studies reported a decrease of MEP amplitude similar to healthy subjects (36, 37), while others reported an increase (19, 38) or no changes (39). Also, in the premovement phase, a significant lack of MEP facilitation after a sustained motor task was shown in fatigued PwMS compared to controls and not-fatigued patients (40, 41), suggesting a disruption of the brain networks involved in motor preparation which has been correlated to structural and functional changes in frontal-thalamic pathways (41).

Different paired-pulse TMS studies have demonstrated, in healthy subjects, physiological modifications of cortical excitability as a result of fatigue development. Paired-pulse TMS protocols are used to test different cortical circuits (42) and include short-interval cortical inhibition (SICI) (43), a protocol related to inhibitory gamma-aminobutyric acid (GABA)-A interneurons, in which a subthreshold conditioning first pulse inhibits the response to a suprathreshold second pulse delivered 1–5 ms later (44); intracortical facilitation (ICF) (45), linked to glutamatergic intracortical circuits in which a subthreshold conditioning first pulse enhances the response to a suprathreshold second pulse delivered 7–20 ms later (46); and late intracortical inhibition (LICI) (47), mediated by GABA-B receptors in which two suprathreshold pulses at long-interstimulus intervals of 50–200 ms are delivered (48). Benwell et al. (49) showed that SICI initially increases and then decreases as force declines during a fatiguing exercise involving the first dorsal interosseous (FDI) muscle. Similarly, Maruyama et al. (50) found a transient reduction of SICI in FDI muscle after isometric contractions, while there was no change in ICF. By contrast, Hunter et al. (51) likewise found a reduction of SICI, while ICF decreased during a sustained submaximal voluntary muscle contraction. Besides that, changes of ICF or SICI seem to depend also on the type of fatiguing motor task used in the experimental protocol—for instance, being different during handwriting compared to isometric finger abduction (52).

In PwMS, different alterations in cortical excitability parameters have been described. Liepert et al. (37) found that, compared to healthy controls and to PwMS without fatigue, SICI was reduced in PwMS with fatigue, already at baseline, before the fatiguing exercise. In contrast, Morgante et al. (40) found similar values of SICI and ICF in PwMS with and without fatigue and in healthy controls, while Chalah et al. found a significant reduction of SICI in non-fatigued compared to fatigued PwMS and no significant difference in ICF and other TMS measures (53).

Another neurophysiological measure which can be assessed through TMS is the cortical silent period (CSP) that is an interruption of the voluntary muscle contraction after a TMS pulse over the contralateral motor cortex and is thought to be mediated by GABA-B inhibitory neurotransmission, (54). CSP duration in PwMS predicted fatigue and was associated with poor cardiovascular fitness (55).

Several studies have investigated the changes of cortical plasticity of PwMS through rTMS protocols (56, 57), but only a few of them have explored their role in fatigue pathogenesis.

Morgante et al. (40) found that PwMS have reduced plasticity demonstrated by the lack of MEP increase after the 5-Hz rTMS protocol, without any difference between fatigued and not-fatigued patients. Conte et al. (58) found instead that, during an attention-demanding task, the response to 5-Hz rTMS and paired associative stimulation (PAS)—a neuromodulatory protocol consisting of repetitive peripheral nerve stimulation combined with TMS over the contralateral motor cortex (59)—significantly differs between PwMS with or without fatigue. Indeed in fatigued patients both PAS and 5-Hz stimulation did not produce the expected changes in cortical excitability, while in not-fatigued patients they both increased the MEP response, although less efficiently than in healthy subjects.

TMS techniques do not allow a complete evaluation of brain subcortical structures, the role of which seems to be crucial in fatigue generation. In a recent study, Capone et al. (60) evaluated how high-frequency oscillations (HFOs)—a burst of fast oscillations that overlies the cortical response of median nerve somatosensory-evoked potentials—are influenced by a fatiguing exercise in a cohort of 15 PwMS and 15 healthy controls. They showed a significant change of the early component of HFOs, reflecting the possible primary role played by the thalamus in the pathogenesis of MS-related fatigue, while the latter component reflects that the cortico-cortical network activity in the somatosensory cortex was not modified significantly. Furthermore, increasing evidence from neuroimaging studies is supporting the hypothesis that the thalamus is a key player in fatigue generation (6).

Fatigue is a complex symptom involving both cognitive and motor domains and multiple factors, in addition to sensorimotor dysfunction as assessed by EEG and EPs, which can contribute to its pathogenesis and/or exacerbate its manifestations (demographics, comorbidity, genetics, diet, exercise, depression, cognitive impairment, pain, and sleep disorders) (6). Neurophysiology can also play an important role in defining and quantifying some of these factors. For instance, event-related potentials (ERP) could be a useful tool to investigate the mechanisms involved in the pathogenesis of cognitive fatigue.

Pokryszko-Dragan et al. found that fatigued PwMS have worse cognitive performances and delayed latency in the P300 component of the auditory ERP and also in the early stage of the disease. These results were confirmed by Chinnadurai et al. (61) in a sample of 50 PwMS using a modified version of auditory ERP. However, a recent study by Lazarevic et al. (62) did not find any effect of depression and fatigue on the ERP parameters. Thus, further research is needed to clarify the role of ERP in the assessment of cognitive impairment in PwMS.

It has been demonstrated that sleep disorders such as obstructive sleep apnea (63), restless leg syndrome (64, 65), periodic limb movements (66), and rapid eye movement behavior disorders (67) are more frequent in PwMS than in the general population and can contribute to the development of motor (2) and cognitive fatigue (68). In all these disorders, overnight polysomnography is essential to make a diagnosis and to quantify the consequent reduction of sleep efficiency (69).

Moreover, the disease itself can produce pathological and functional modifications in the CNS that alter the restorative sleep capacity and thus exacerbate fatigue perception. This phenomenon was investigated by Bridoux et al. using TMS for assessing the reduction of MEP amplitude induced by an exercise (post-exercise corticomotor depression or PECD). They demonstrated that, in healthy subjects, sleep enhances recovery from PECD, while in PwMS, the restorative effect of sleep is reduced or lost (70).

Autonomic dysfunction is very common among PwMS and can occur since the earliest stages of the disease. It is mainly caused by demyelinating lesions located in the periventricular region of the fourth ventricle, in the brainstem, and in the spinal cord (69). Autonomic dysfunction can produce different symptoms affecting the bowel, the bladder, the heart, and the blood vessels.

The functionality of the autonomic nervous system can be tested by the Quantitative Sudomotor Axon Reflex testing (71) and the study of cardiovascular parameters such as blood pressure and heart rate response to Valsalva maneuver, heart rate variability during deep breathing, and blood pressure and heart rate changes during tilt test (72).

In particular, cardiac autonomic dysfunction has been associated to fatigue in PwMS (73), but the mechanisms and significance of this association remain unclear.

Some authors have hypothesized that MS-related fatigue is caused by a sympathetic vasomotor dysfunction with a normal parasympathetic activity (74–76).

On the contrary, other studies found that fatigued PwMS have a reduction in vagal activity compared to controls (77–79).

Recent evidence suggests that pupillometry could be an alternative method to evaluate the involvement of the autonomic nervous system in PwMS. Indeed the pupil size depends on the balance between the sympathetic and parasympathetic components of the autonomic nervous system. For instance, de Rodez Benavent et al. (80) investigated the changes in pupil size during problem-solving in MS patients (with and without fatigue) vs. controls. They found that MS-related changes in cognition and fatigue could be associated with changes in the autonomic regulation of task-related pupillary responses.

Taken together, the neurophysiologic data demonstrated that MS-related fatigue seems to have a central origin. The changes in EMG parameters, described in MS patients (32, 33), are thought to be more a consequence of alterations in CNS structures rather than a primary determinant of fatigue. However, it cannot completely be ruled out that such changes could be the epiphenomenon of peripheral alterations occurring at the neuromuscular level.

Neuroimaging studies (60, 81, 82) demonstrated that the main pathogenic substrate of MS-related fatigue could be a dysfunction of the circuits between the thalamus, the basal ganglia, and the cortex, and neurophysiological findings support this hypothesis. Indeed single-pulse TMS studies demonstrated that in MS patients the pathogenesis of fatigue is not driven by mechanisms directly related to corticospinal functioning but is due to alterations in structures located upstream to the primary motor cortex (39). In particular, both EEG (34) and TMS studies (37, 40, 58) pointed out the role of cortical areas involved in movement preparation and attention. For instance, Sandroni et al. (83) found that, in PwMS, fatiguing tasks are associated with a change in ERP without significant modifications in MEP parameters, thus suggesting that fatigue affects neural processes acting after stimulus evaluation and before the activation of the primary motor cortex.

More recently, Capone et al. (60) explored the contribution of the thalamus by means of HFOs obtained from the median nerve SEP, demonstrating that a dysfunction of the thalamo-cortical axons contributes to fatigability in MS patients.

Although CNS functional alterations are consistently reported by neurophysiological studies, their significance remains largely unknown because they were considered by some authors as pathogenic factors (40) and by others as the epiphenomena of adaptive processes (60). According to the first hypothesis, neurophysiologic techniques measure the change in the activity of CNS networks caused by the MS-related damage of gray and white matter. On the other side, according to the alternative hypothesis, this damage produces compensatory/adaptive mechanisms that can be recorded by means of neurophysiological techniques.

More broadly, several structural and functional abnormalities in various cortico-subcortical neural networks (e.g., fronto-striatal network, cortico-striato-thalamo-cortical loop) occur during MS as a result of inflammation, neurodegeneration, and compensatory neuroplasticity processes. From this perspective, the development of fatigue could depend on the dynamic balance between damage and restorative processes during the disease's course (8). Indeed the latter can be predominant in the initial phase of the disease, thus masking the clinical occurrence of fatigue, while, later on, the damage could prevail so that patients experience clinically relevant fatigue. Accordingly, the heterogeneity in the results of neurophysiological studies can depend on the stage of the disease in which the recording has been done.

Interestingly, the neurophysiological markers of fatigue at different levels, such as changes in EMG parameters (33), in HFO features (60), or in cortical plasticity (40), can also be observed in MS patients without fatigue. This finding could suggest that an impairment in fatigability mechanisms (expressed by neurophysiological alterations) does exist in MS since the earliest phases of the disease, independently from the level of fatigue in everyday life measured through questionnaires. This is not surprising because fatigue is a multifactorial and complex symptom, and different factors, in addition to thalamo-cortical dysfunction, could be necessary to make it clinically relevant.

MS can cause extensive damage of the CNS, so it is not surprising that autonomic nervous system involvement or subtle alterations of cognitive functioning may occur at any stage of the disease. Thus, these are other factors that need to be considered as potential players in fatigue generation, but evidences are not unambiguous. Sleep disorders should also be taken into account since the impairment of a restorative process can exacerbate—or even be one of the main generators— fatigue (2, 68, 70).

Longitudinal studies involving patients at different stages of the disease (from clinically isolated syndrome to advanced progressive MS) and investigating possible factors involved in fatigue perception (such as genetics, comorbidity, cognitive impairment, depression, and sleep disorders) could contribute to corroborate such hypothesis. In Table 1, we have summarized the studies that have used neurophysiological techniques for investigating fatigue pathogenesis.

**Table 1:** Neurophysiological studies exploring the pathogenic mechanisms of fatigue in PwMS.

**References**	**Neurophysiologic technique**	**Sample size**	**Sample composition**	**Main findings**
Steens et al. (22)	EMG	20 PwMS + 20 HCs	20 patients (RR); age range: 20–58 years; EDSS <5.5	Positive correlation between fatigue perception and the decline of MCV during a sustained contraction
Rice et al. (17)	EMG	4 PwMS + 16 HCs	4 patients (SP, RR); age range: 28–53 years; mean EDSS 4.6	PwMS present lower values of MVC
Sheean et al. (18)	EMG	21 PwMS + 19 HCs	21 patients (RR; SP, PP); age range: 26–55 years; mean EDSS: 2–8	PwMS present lower values of MVC
Perretti et al. (19)	MEP	41 PwMS	41 patients (RR), on IFN b1a treatment; age range: 30.7 ± 8.8; EDSS: 3.2 ± 0.5; divided into fatigued and not-fatigued	MS patients do not have TMS MEP depression following fatiguing exercise, while post-exercise MEP facilitation was similar to that seen in normal subjects
Steens et al. (22)	EMG	20 PwMS + 20 HCs	20 patients; age range: 21–58 years; EDSS ≤ 5	Decrease of voluntary activation during fatiguing exercise in PwMS in comparison to HC
Wolkorte et al. (23)	EMG	45 PwMS + 25 HCs	45 patients (RR, SP); age range: 20–65 years; EDSS: 0–7	Compared to controls, the SPMS patients had reduced voluntary activation during brief and sustained contractions.
Eken et al. (32)	EMG	8 PwMS + 10 HCs	8 patients (RR, SP, PP); age range: 49 ± 9 years; EDSS: 1–6	Prolonged walking produces a significant decrease of EMG median frequency and an increase of root mean square EMG signal of the soleus muscle
Severijns et al. (33)	EMG	16 PwMS + 16 HCs	16 patients (RR, SP, PP); age range: 55 ± 8 years; mean EDSS: 6; divided into fatigued and not-fatigued	PwMS with hand grip weakness, experience a larger increase in fatigue compared to PwMS with normal hand grip strength
Leocani et al. (34)	EEG	33 PwMS + 14 HCs	33 patients; EDSS <1.5; divided into fatigued (age: 33 ± 8 years) and not-fatigued (age: 32 ± 6 years)	In PwMS, FSS correlated positively with ERD over midline frontal structures during movement and inversely with contralateral sensorimotor ERD after movement
Petajan and White (36)	MEP	32 PwMS + 10 HCs	32 patients; divided into 2 subgroups: patients without weakness of upper limbs (age: 44 ± 10.3 years) and patients with weakness of upper limbs (age: 42.9 ± 9.9 years)	Decrease of MEP amplitude similar to HCs
Liepert et al. (37)	MEP	16 PwMS RR + 6 HCs	16 patients, divided in 2 subgroups: fatigued (FSS > 4, mean EDSS: 3.1); not-fatigued (FSS <4, mean EDSS: 2.9)	Decrease of MEP amplitude similar to HC; in fatigued patients, SICI was reduced at baseline
Thickbroom et al. (38)	MEP	10 PwMS + 13 HCs	10 patients (RR); age range: 33–64 years; EDSS ≤ 4; MRC grade ≥ 4/5	Increase of MEP amplitude in PwMS compared to HC
Mordillo-Mateos et al. (39)	MEP	17 PwMS + 16 HCs	17 patients (RR; SP); mean age: 36.3 ± 9.5 years; mean EDSS: 5	No changes in MEP amplitude in the two groups
Morgante et al. (40)	MEP	33 PwMS	33 patients (RR), divided into 2 subgroups: fatigued (mean age 38 ± 9.4 years; mean EDSS 1.6 ± 0.6) and not-fatigued (mean age 41.1 ± 10.9 years; mean EDSS 1.8 ± 0.6)	PwMS with fatigue lacked pre-movement facilitation compared to PwMS without fatigue and HC
Conte et al. (58)	5Hz rTMS, PAS	25 PwMS + 18 HCs	25 patients (RR); EDSS <3.5; divided into 2 subgroups, fatigued (mean age 41.3 ± 7.7 years; mean EDSS 1) and not-fatigued (mean age 38.3 ± 8.4 years; mean EDSS 1.1)	In non-fatigued patients, PAS and rTMS increased the MEP response; in fatigued patients, they did not produce changes in cortical excitability
Capone et al. (60)	SEP, HFO	15 PwMS + 15 HCs	15 patients (RR); mean age: 42.1 years; mean EDSS 1	Fatiguing task induces a change in the early component of HFOs in PwMS
Russo et al. (41)	MEP	24 PwMS + 10 HCs	24 patients (RR), age range: 18–65 years; EDSS ≤ 2.5	Premovement facilitation is reduced in fatigued PwMS
Russo et al. (84)	MEP	30 PwMS	30 patients (RR); mean age: 24–63 years; EDSS <3.5; divided into 2 subgroups, fatigued and not-fatigued	Fatigue is associated with a disruption of brain networks involved in motor preparation processes, depending on frontal-thalamic pathways
Chalah et al. (53)	MEP	38 PwMS	38 patients (RR, PP, SP); age range: 34–67 years; EDSS: 3–6.5; divided into 2 subgroups, fatigued and not-fatigued	Fatigued patients had higher depression, anxiety, alexithymia scores, higher SICI, larger caudate nuclei, and smaller left parietal cortex.
Chaves et al. (55)	MEP	82 PwMS	92 patients (RR; PP, SP); mean age: 47.40 ± 10.2 years; EDSS 2.04 ± 1.	Longer CSP predicted worsened fatigue in PwMS
Pokryszko-Dragan et al. (85)	ERP	86 PwMS + 40 HCs	86 patients (CIS; RR; SP); age range: 19–60 years; EDSS: 1–6.5; divided into 3 groups: not fatigued, moderately fatigued, severely fatigued	Fatigued PwMS have worse cognitive performances and delayed latency in P300 component of auditory ERP
Pokryszko-Dragan et al. (86)	ERP	44 CIS + 45 HCs	44 patients (CIS); age range: 21 – 48 years; EDSS: 1–2	N200 latency was correlated with fatigue.
Chinnadurai et al. (61)	ERP	50 PwMS + 50 HCs	50 patients (RR; PP; SP); age range: 13–66 years; EDSS: 1–9	Clinical measures of cognitive fatigue were correlated with the neurophysiological measures (ERP)
Lazarevic et al. (62)	ERP	81 PwMS + 32 HCs	81 patients (RR); age: 41.09 ± 8.72 years; EDSS: 0–7; divided in two groups: fatigued and not fatigued	Depression and fatigue have no effect on ERP amplitude and latency
Bridoux et al. (70)	MEP	30 PwMS + 15 HCs	12 fatigued patients (RR; SP); mean age: 44 ± 3 years; EDSS: 1–3.5	In PwMS, sleep does not enhance motor recovery from PECD following a fatiguing exercise
Lebre et al. (74)	ANS testing	50 PwMS	50 patients (RR); mean age 37 years; EDSS <3.5; divided in two subgroups: fatigued and not-fatigued	Loss in the capacity to increase the blood pressure in patients with fatigue, suggesting a sympathetic dysfunction
Flachenecker et al. (75)	ANS testing	60 PwMS + 36 HCs	60 patients (RR); mean age 41.5 ± 9.9 years; mean EDSS 3.0; divided in two subgroups: fatigued and not-fatigued	The median HR response to standing (HR-Post30/15) was significantly reduced, and BP-Grip tended to be lower in pwMS compared to HCs.
Niepel et al. (76)	Sleep study	26 PwMS + 9 HCs	26 patients (RR; SP; PP); divided in 2 subgroups, fatigued (FSS > 5; age range 49.4 ± 9.2 years) and not-fatigued patients (FSS <4.0; age range 41.8 ± 13.1 years)	Fatigue patients showed evidence of reduced level of alertness on a number of subjective and objective measures of alertness, in contrast to non-fatigued MS patients and HCs
Keselbrener et al. (77)	ANS testing	10 PwMS + 10 HCs	10 patients; age: 22–58 years; FSS > 3.5	Fatigued PwMS showed a reduction in vagal activity which was more marked than in the control subjects
Heesen et al. (78)	ANS testing	23 PwMS + 25 HCs	23 patients (RR; SP); mean age: 40.13 ± 2.23 years; mean EDSS 2.36 ± 0.36. 14 patients on DMD (8 interferon, 5 glatiramer acetate, 1 azathioprine)	Cognitive stress induces IFNγ production in HC but not in MS patients with fatigue. Reduced cardiac response might indicate an autonomic dysfunction in PwMS.
Sander et al. (79)	ANS testing	53 PwMS	53 patients (RR, SP, PP); mean age: 50.1 ± 8.7 years; mean EDSS 3.3 ± 1.7	Reduced responsiveness and high- and very-low-frequency components of HR variability, indicating an increased parasympathetic activity
de Rodez Benavent et al. (80)	ANS (pupillary response)	49 PwMS + 46 HCs	49 patients (RR); age range: 18–50 years; mean EDSS 1.9 ± 0.8	MS-related changes in cognition and fatigue could be associated with changes in the autonomic regulation of task-related pupillary responses

*EMG, electromyography; PwMS, people with multiple sclerosis; HCs, healthy controls; RR, relapsing–remitting; PP, primary progressive; SP, secondary progressive; EDSS, expanded disability status scale; MVC, maximum voluntary contraction; MEP, motor-evoked potentials; IFN, interferon; TMS, transcranial magnetic stimulation; EEG, electroencephalography; ERD, event-related desynchronization; FSS, fatigue severity scale; SICI, short-interval intracortical inhibition; PAS, paired associative stimulation; rTMS, repetitive transcranial magnetic stimulation; SEP, somatosensory-evoked potentials; HFO, high-frequency oscillations; CSP, cortical silent period; CIS, clinically isolated syndrome; PECD, post-exercise cortical depression; ERP, event-related potentials; ANS, autonomic nervous system; HR, heart rate; BP, blood pressure; MS, multiple sclerosis*.

## Neurophysiology for Monitoring the Response to Therapies for Fatigue

The most frequently used pharmacological treatments for fatigue are amantadine, 4-aminopyridine, and modafinil. The non-pharmacological interventions include physical (e.g., aerobic exercises, resistance training, yoga, and tai-chi) and psychological/cognitive approaches (e.g., cognitive behavioral therapy, education programs, and mindfulness interventions). However, evidence supporting the efficacy of these interventions is still preliminary and, sometimes, conflicting (87).

Amantadine is an antiviral agent firstly introduced to prevent and treat flu viruses. Animal models have shown that amantadine induces the release of dopamine from nerve endings (88). Moreover, one clinical trial has shown an increased level of beta-endorphin and beta-lipoprotein after amantadine assumption, with clear clinical improvement (89). The real mechanism of action of amantadine as fatigue therapy is not yet clear, but the fact that amantadine acts as a dopaminergic factor supports the dopamine imbalance theory for fatigue generation (90). One relevant study, addressing the neurophysiological effects of amantadine in MS-related fatigue, was conducted by Santarnecchi et al. (91). They found that chronic treatment with this drug improves clinical fatigue (assessed through questionnaires) and restores GABAergic inhibitory mechanisms in the motor cortex of PwMS, as indicated by the normalization of CSP in basal condition and by the reduction of CSP duration after a fatiguing task. Reis et al. (92) evaluated the effect of a single dose of amantadine on human motor cortex excitability in healthy subjects. They showed that a single dose of amantadine significantly decreases ICF and increases LICI in the motor cortex. MEP recruitment curves, motor thresholds, and duration of CSP remained unchanged after treatment. These data suggested that a single dose of amantadine is able to modulate motor cortex excitability, possibly involving GABAergic and glutamatergic neurotransmission.

Another drug, tested for MS-related fatigue, was modafinil, a central alpha-adrenergic agonist approved for the treatment of attention-deficit hyperactivity disorder and narcolepsy. Lange et al. (93) reported a significant improvement of fatigue questionnaire scores and in the nine-hole peg test, after modafinil administration, in a group of 21 PwMS. Furthermore, they tested different TMS protocols before and after 8 weeks of treatment, showing an increase of MEP size by paired pulse TMS, in the modafinil group.

Nagels et al. (94) evaluated visual- and auditory-evoked potentials (EP) for predicting the response to modafinil treatment (100 mg, once daily, for 4 weeks), in 33 PwMS with fatigue. They found that the latency of auditory P300 predicted the treatment response with a good specificity and sensitivity. In particular, a shorter latency at baseline was associated with a better response to modafinil treatment.

In order to better clarify the mechanisms of action of modafinil in fatigue relief, Niepel et al. (76) investigated the effect of a single dose (200 mg) of modafinil on measures of alertness and autonomic function in fatigued PwMS compared to not-fatigued PwMS and healthy controls.

They found that fatigued patients had a reduced level of alertness and cardiovascular sympathetic activation compared to the other two groups, and modafinil was able to reverse these deficiencies. On the basis of these findings, they hypothesized that the anti-fatigue effect of modafinil was related to the activation of the noradrenergic locus coeruleus (76).

Despite these interesting data, at present, there is no indication, in clinical practice, for the use of modafinil for fatigue relief.

Potassium channel blockers—e.g., 4-aminopyridine (4-AP)—belong to a group of drugs able to restore conduction propriety in demyelinating axons as shown in animal models (95). Different trials have also explored the central effect of 4-AP, speculating on a potential role in optimizing neurotransmitter release at the synaptic level (dopamine, acetylcholine, noradrenaline, and serotonin). This latter hypothesis is supported by the observation of an increase BOLD signal during a motor task following a 3,4-diaminopyridine administration compared with a placebo dose assumption (96).

Sheean et al. (97) evaluated changes in TMS-evoked corticospinal excitability parameters in eight PwMS with fatigue before and after treatment with 3,4-diaminopyridine. The motor performance of adductor pollicis muscle was evaluated by TMS, rapid voluntary movements, and a fatiguing exercise test consisting of a sustained isometric contraction. After 3 weeks, fatigue was significantly reduced but neurophysiological parameters (central motor conduction time and MEP amplitude) did not change in the treated patients compared to the untreated ones. These findings suggest that the effect of 3,4-diaminopyridine on fatigue could be linked with mechanisms and structures other than corticospinal tract functionality. Moreover, methodological factors should be considered in the interpretation of these results. Indeed only upper limbs spared from the disease were evaluated, thus representing a major limitation of the study.

More recently, Marion et al. designed a randomized double-blind placebo-controlled trial to investigate the effect of modified-release 4-aminopyridine (fampridine) on upper limb function, fatigue, and several neurophysiological parameters such as visual-evoked potentials (latency and amplitude), somatosensory-evoked potentials (latency and amplitude), motor-evoked potentials (latency), central motor conduction time, resting motor threshold, MEP recruitment curves, and paired-pulse TMS protocols. They found that fampridine (10 mg bd, for eight consecutive weeks) did not produce significant changes in upper limb function, fatigue, and neurophysiological parameters (98).

Over the last years, various studies have demonstrated that neurophysiology can be helpful in measuring and predicting response to treatment. However, the results are not definitive since data are scarce and sometimes not conclusive. Studies greatly differ from each other in variables such as outcome measures, treatment and follow-up duration, neurophysiological techniques, and clinical features of patients. Moreover, to the best of our knowledge, no study has evaluated, through neurophysiological tools, the effectiveness of non-pharmacological interventions such as physical, psychological, and cognitive approaches. Anyway, it still seems reasonable to assume that neurophysiology can have a role in monitoring the response to fatigue treatment, and more studies on the matter are warranted.

In Table 2, we have summarized the studies that have used neurophysiological techniques for monitoring the treatment for fatigue in PwMS.

**Table 2:** Neurophysiological studies for monitoring response to therapies for fatigue in PwMS.

**References**	**Therapy**	**Neurophysiologic technique**	**Sample size**	**Sample composition**	**Main findings**
Santarnecchi et al. (91)	Amantadine	MEP, EMG for CSP study	10 PwMS + 10 HCs	10 patients (RR; SP); age range: 24–44 years; mean EDSS: 2.1 ± 1.4	Normalization of CSP in basal condition and a reduction of CSP duration after the fatiguing task
Reis et al. (92)	Amantadine, single dose	MEP, EMG for CSP, SICI, LICI	14 HCs	14 healthy volunteers; mean age: 25 ± 2.8 years	A single dose of amantadine was able to modulate motor cortex excitability (decreases ICF and increases LICI in M1)
Lange et al. (93)	Modafinil, 100 mg/day for the first week and 200 mg/day for subsequent 7 weeks vs. placebo	MEP	21 PwMS	21 patients, FSS ≥ 36, EDSS <7.0; divided into 2 subgroups: treated (mean age: 42.6 ± 9.7 years; mean EDSS; 3.1 ± 0.6) and placebo (mean age: 44.1 ± 12.1 years; mean EDSS: 3.2 ± 1.1)	Increase MEP size by paired pulse TMS in the modafinil group
Nagels et al. (94)	Modafinil, 100 mg, once daily, for 4 weeks	ERP	33 PwMS	33 fatigued patients (RR; SP; PP); mean age: 43 ± 2 years; mean EDSS: 5	A shorter P300 latency at baseline was associated with a better response to modafinil treatment
Sheean et al. (97)	3,4- diaminopyridine	MEP	8 PwMS	8 patients (RR; SP; PP); mean age: 39 years; mean EDSS: 6	After treatment, fatigue was significantly reduced but the neurophysiological parameters (central motor conduction tip and MEP amplitude) did not change
	4-AP vs. fluoxetine	SEP, MEP	60 PwMS	60 patients (RR); age range: 18–50 years; mean EDSS: 5.5; divided into 2 subgroups: fatigued (mean EDSS: 3.3 ± 2.5) and not-fatigued (mean EDSS: 3.1 ± 2.3)	Significant reduction of the fatigue questionnaire scores, with a greater reduction for the 4-AP subgroup
Marion et al. (98)	4-aminopyridine, 10 mg bd, for 8 consecutive weeks vs. placebo	VEP, SEP, MEP	40 PwMS	40 patients (RR; SP; PP); mean age: 52 years; mean EDSS: 6.0	Fampridine did not produce significant changes in upper limb function, fatigue, and neurophysiological parameters

*MEP, motor evoked potentials; EMG, electromyography; CSP, cortical silent period; PwMS, people with multiple sclerosis; HCs, healthy controls; RR, relapsing–remitting; PP, primary progressive; SP, secondary progressive; EDSS, expanded disability status scale; SICI, short interval intracortical inhibition; LICI, long-interval intracortical inhibition; TMS, transcranial magnetic stimulation; ERP, event-related potentials; SEP, somatosensory-evoked potentials; VEP, visual-evoked potentials*.

## Neurophysiology as Innovative Treatment for Fatigue in MS Patients

Neurophysiological studies are being carried out not only to identify objective and measurable markers of fatigue, as previously illustrated, but also to find neuromodulation protocols able to reduce this disabling symptom.

NIBS approaches are playing a major role in this research setting, following a large neurophysiological evidence of central abnormalities in PwMS with fatigue (34, 39, 56, 99).

In Table 1, the results of a MEDLINE research on sham-controlled NIBS studies for the treatment of fatigue in PwMS is presented, and the stimulation parameters are described for each study.

Transcranial direct current stimulation (tDCS) is the NIBS technique mostly used so far (cf. Table 1). It is classically assumed that tDCS can modulate human brain activity with effects that could outlast the period of stimulation by inducing a subthreshold shift of the resting membrane potential toward depolarization (anodal tDCS) or hyperpolarization (cathodal tDCS) (15). Beyond local effects, connectional (axonal) and non-neuronal effects have also been described (15). The tDCS mechanisms of action are still incompletely understood; an effect on calcium-dependent synaptic plasticity of glutamatergic neurons and a local reduction in GABA neurotransmission have been hypothesized (15).

Anodal tDCS applied to the motor cortical areas reduced motor fatigue in healthy subjects (100, 101). In patients with MS-related fatigue, anodal tDCS has been used with variable effects, depending on the parameters of stimulation and the clinical characteristics of the patients included in the studies.

As shown in Table 1, different targets have been stimulated by anodal tDCS. The evidence of functional alterations in the frontal areas in PwMS with fatigue (8) focused the attention of some researchers on the stimulation of the left dorsolateral prefrontal cortex (DLPFC).

Among these studies, negative results were reported by Saiote et al. (102) and Ayache et al. (103). Some methodological factors such as the wash-out duration and the stimulation intensity (102), the stimulation duration, and the heterogeneity of the population included (103) could have played a role in these results. Other three studies reported positive results on fatigue after anodal tDCS was applied over the left DLPFC (104–106). Among these, worthy of note are the use of a remotely supervised tDCS system in combination with a computer-based cognitive training (105) and the use of objective outcome measures, such as the P300 evoked potential and the reaction time (106). The application of anodal tDCS to the motor cortex bilaterally (107) and to the right parietal cortex (108) also gave a preliminary evidence of efficacy.

The group of Tecchio et al. focused on a personalized anodal tDCS approach targeting the whole-body primary somatosensory areas (S1) bilaterally, following the evidence of S1 reduced excitability and M1 hyperexcitability in PwMS with fatigue (109–112). They used a tailored procedure with personalized electrodes based on the patients' brain MRI located in place through an MRI-guided neuronavigation system. In a more recent study of this group, the importance of the individual baseline neural networks activity has been outlined as a further parameter for individualized treatment (110). The results of their studies support the efficacy of personalized tDCS approaches.

Only one sham-controlled study has explored the effects on MS-related fatigue of another NIBS technique called transcranial random-noise stimulation (tRNS). This stimulation was applied on frontal regions but produced negative results (113).

The other NIBS technique introduced in the research setting for the treatment of fatigue in PwMS is TMS. (114). Repetitive protocols of TMS showed long-lasting effects on cortical excitability in patients with stroke (115), MS-related spasticity (116) and major depression (13).

Regarding MS-related fatigue, three sham-controlled studies using TMS showed promising results (117–119). Two of these studies used TMS in combination with physical therapy and enrolled patients affected by spasticity (118, 119). Different TMS protocols were used: intermittent theta-burst stimulation (iTBS) applied to the M1 leg area (119), deep TMS, delivered with specific H-coils to the left prefrontal cortex and to bilateral M1 (117), and 20-Hz repetitive TMS and iTBS applied to bilateral M1 (118). Preliminary evidence of efficacy was described for all the protocols excepted for iTBS on bilateral M1 (118).

In a recent systematic meta-analysis, Liu et al. reviewed the efficacy and safety of NIBS specifically for the treatment of MS-related fatigue (120). They performed a literature search for sham-controlled brain stimulation studies based on tDCS, rTMS, tRNS, and transcranial alternating current stimulation (tACS). A total of 14 eligible studies published from 2011 to 2018, for a total of 207 MS patients, were found: 11 tDCS studies, one rTMS study, one iTBS (combined with exercise therapy) study, and one tRNS study. A significant improvement in fatigue scores compared to sham was found after tDCS treatment. A subgroup analysis demonstrated significance for the intensity of 1.5 mA and for bilateral S1 stimulation location. The two TMS studies and the tRNS study did not reach statistical significance.

Several data are available about the therapeutic use of NIBS for reducing MS-related fatigue (Table 3). These techniques—and in particular tDCS and some TMS protocols—have shown to be effective as add-on therapy for fatigue management, and more studies are needed to explore their further implementation. The mechanisms by which NIBS could improve fatigue are still unclear (8, 15, 104). Different hypothesis have been proposed such as presynaptic increase of spinal drive from motor cortex, modulation of premotor areas, increase in motivation, decrease in muscle pain, increase in muscle coupling, promotion of changes in cortical resting state activity and cortico-cortical connectivity, and induction of long-term potentiation-like and long-term depression-like neuroplastic changes at a local and/or network level. The potential role of altered oscillatory activity in the pathogenesis of MS-related cognitive fatigue and the potential advantage of tACS application have also been outlined (122). A better comprehension of the pathogenesis could be useful to develop therapies that specifically target the mechanisms of fatigue generation in MS.

**Table 3:** Sham-controlled NIBS studies for the treatment of MS-related fatigue.

**References**	**Anode: location dimensions**	**Cathode: location dimensions**	**Stim duration stim intensity**	**Efficacy**	**Fatigue evaluation methods**	**Study design**	**Sample size and MS subtype**	**Sample composition (values expressed as mean ± SD, when available)**	**Adverse effects**
**1) Anodal Tdcs**									
Saiote et al. (102)	Left DLPFC 5 × 7 cm	Right forehead 6 × 15 cm	20 min/day, 5 days 1 mA	No	- FSS - MSFSS - MFIS	Crossover, sham-controlled (2-week wash-out)	13 RR	Clinically definite MS (121) Age: 46.9 ± 6.8 EDSS: 3.5 ± 4.0 FSS: 5.67 ± 2.47 MFIS: 47 ± 31	Tingling, light headache
Ayache et al. (103)	Left DLPFC 25 cm^2^	Right supraorbital region 25 cm^2^	20 min/day, 3 days 2 mA	No	MFIS (secondary outcome)	Crossover, sham-controlled (3-week wash-out)	16 (11RR, 4SP, 1PP)	Clinically definite MS (121) and history of neuropathic pain with VAS >40 Age: 48.9 ± 10 EDSS: 4.25 ± 1.4 MFIS: 52.6 ± 12.2	Insomnia, nausea, severe headache, phosphenes
Chalah et al. (104)	a) Left DLPFC 25 cm^2^ b) Right PPC 25 cm^2^ in different blocks	a) Right supraorbital region 25 cm^2^ b) Cz (EEG 10-20 system) 25 cm^2^	20 min/day, 5 days 2 mA	a) Yes (on FSS and on MFIS physical and psychosocial subscales) b) No	- FSS - MFIS - VAS	Crossover. sham-controlled (3-week wash-out)	10 (9 RR, 1 SP)	Clinically definite MS (121) Age: 40.50 ± 11.18 EDSS: 2.3 ± 2.5 FSS: 6.5 ± 3.8	a) None b) Insomnia, headache
Charvet et al. (105)	Left DLPFC 5 × 5 cm	Right DLPFC 5 × 5 cm	Remotely supervised tDCS combined with computer-based cognitive training 20 min/day, 20 days over 4 weeks From 1.5 to 2 mA	YES	- FSS - PROMIS-fatigue short form - VAS	Randomized, sham-controlled	27 (15 active of which 40% RR, 12 sham of which 58% RR)	Clinically definite MS Active group (*n* = 15): - age: 44.8 ± 16.2 - EDSS: 6.0 (range 0.0–7.0) - FSS (%clinical fatigue): 50 Sham group (*n* = 12): - age: 43.4 ± 16.2 - EDSS: 3.5 (range 0.0–8.5) - FSS (%clinical fatigue): 76	Tingling, itching, burning, head pain, difficulty concentrating
Fiene et al. (106)	Left DLPFC 5 × 5 cm	Right shoulder 5 × 7 cm	Single session of 27.29 ± 1.15 min (10 min tDCS only, 20 min tDCS during testing) 1.5 mA	Yes (on P300 amplitude and RT, not on subjective fatigue)	- P300 amplitude and latency during an auditory oddball task - simple RT in an alertness test	Crossover, sham-controlled (1-week wash-out)	15 (14 RR, 1 SP)	Clinically definite MS (121) with a minimum of 9 points on the cognitive subscale of the WEIMuS age: 43.20 ± 14.97 EDSS: 3.54 ± 1.94 WEIMuS physical: 19.73 ± 5.70	itching
					- subjective fatigue via a 10-point numerical rating scale and objective fatigue (e.g., WEIMuS physical)				
Ferrucci et al. (107)	Bilateral motor cortex 5 × 7 cm	Right deltoid 5 × 7 cm	15 min/day, 5 days 1.5 mA	Yes (n23, 15 responders)	FIS	Crossover, sham controlled (1-month wash-out)	25 (22 RR, 3 SP)	Clinically definite MS (121) Responders (*n* = 15): - age: 40.3 ± 2.3 - EDSS: 3 ± 0.4 - FIS anodal: 59.5 ± 7.1 - FIS sham: 49.8 ± 7 Non-responders (*n* = 8): - age: 52.5 ± 4.1 - EDSS: 3.8 ± 0.7 - FIS anodal: 58.5 ± 10.7 - FISsham: 61 ± 11.4	Skin reaction
Tecchio et al. (112)	Whole-body bilateral somatosensory cortex Custom-sized S1 electrode using individual brain MRI data 35 cm^2^	Oz (EEG 10–20 system) 7 × 10 cm	15 min/day, 5 days 1.5 mA	Yes	MFIS	Crossover, sham-controlled (washout individually calculated by MFIS compared to baseline)	10 (7 RR, 1 SP, 2 PP)	MS in a mild state (EDSS <3.5) with MFIS > 38 age: 45.8 ± 7.6 EDSS: 1.5 (range 0–3.5) MFIS: 41.6 ± 6.4	None reported
Tecchio et al. (111)		Oz (EEG 10–20 system) 6 × 14 cm	15 min/day, 5 days 1.5 mA	Yes	MFIS	Crossover, sham-controlled (washout individually calculated by MFIS compared to baseline	13 RR	MS patients with physical items mFIS score > 15 age: 45.8 ± 7.6 EDSS: 1.5 (range 0–3.5) MFIS: 41.6 ± 7.5	None reported
Cancelli et al. (109)		Oz (EEG 10–20 system) 7 × 10 cm	15 min/day, 5 days 1.5 mA	Yes	MFIS	Crossover, sham-controlled	10 RR	MS patients (121) with physical items mFIS score > 35 Age: 43.2 ± 13.1	None
								EDSS: 0.9 (range 0–3.5) MFIS: 46.6 ± 15.9	
Porcaro et al. (110)		Oz (EEG 10–20 system) 7 × 10 cm	15 min/day, 5 days 1.5 mA	Yes	MFIS	Crossover, sham controlled (washout individually calculated by MFIS compared to baseline	18 RR	MS patients with EDSS <3.5 and mFIS score > 30 Age: 44.5 ± 10.4 EDSS: 1.1 (range 0–3.5) MFIS pre-real: 45.6 ± 31.66 MFIS pre sham: 44.9 ± 30.67	None reported
Tecchio et al. (111)	Bilateral sensorimotor hand area 70 m^2^	Under the chin 84 cm^2^	15 min/day, 5 days 1.5 mA	No	MFIS	Crossover, sham-controlled (washout individually calculated by MFIS compared to baseline)	8 RR	MS patients with physical items mFIS score > 15 age: 38.1 ± 9.8 EDSS: 2 (range 1–2.5) MFIS: 57.1 ± 19.9	None reported
Hanken et al. (108)	Right parietal cortex (P4) 5 × 7 cm	Right forehead 6 × 15 cm	Single session 20 min	Yes (RT) No (subjective fatigue) Only in subgroup with mild to moderate cognitive fatigue	- RT during a vigilance task - subjective fatigue (VAS)	Randomized, sham-controlled	46 (18 RR, 28 SP) analyzed 20 for each arm, divided in subgroups according to cognitive fatigue assessed by FSMC	MS patients (121) Mild/moderate CF active: - age: 51.8 ± 9.9 - EDSS: 4.0 ± 1.5 Severe CF active: - age: 50.9 ± 8.8 - EDSS: 4.8 ± 1.2 Mild/moderate CF sham: - age: 47.1 ± 10.3 - EDSS: 3.4 ± 2.1 Severe CF sham: - age: 46.5 ± 9.1 - EDSS: 4.5 ± 1.0	None reported
**References**	**Stimulation location**	**TMS protocol TMS coil**	**Stimulation parameters**	**Efficacy**	**Fatigue evaluation methods**	**Study design**	**Sample size and MS subtype**	**Sample composition (values expressed as mean** **±** **SD, when available)**	**Adverse effects**
**2) TMS**									
Mori et al. (119)	M1 leg area contralateral to the affected limb	iTBS + individualized ET (2 h/day for 2 weeks)	1 session/day for 10 sessions over 2 weeks	Yes (real iTBS + exercise therapy group)	FSS Seconday outcome	Randomized, sham-controlled	20 RR	Definite RR MS (121) patients with spasticity affecting predominantly one lower limb Active	Treatment was generally well-tolerated.
			10 bursts of 3 stimuli at 50 Hz, repeated at 5 Hz every 10 s, for a total of 600 stimuli; biphasic waveform 80% AMT					iTBS + ET (*n* = 10): - age: 38.1 ± 10.7 - EDSS: 3.6 ± 1.2 - FSS: 39.5 ± 4.2 Sham iTBS + ET (*n* = 10): - age: 37.7 ± 12.3 - EDSS: 3.8 ± 1.6 Active iTBS only (*n* = 10): - age: 38.3 ± 11.9 - EDSS: 3.5 ± 1.0	
Gaede et al. (117)	a) left PFC (sham-controlled) b) bilateral M1	Deep TMS a) H6-coil b) H10-coil (bihemispherical stimulation)	18 sessions (3/week) over 6 weeks a) 50 bursts of 36 stimuli, 18 Hz, 120% RMT, ITI 20 s,18 min b) 40 bursts of 20 stimuli, 5 Hz, ITI 20 s, 90% RMT, 16 min	Yes (more pronounced for bilateral M1)	FSS	Randomized, sham-controlled	9 PCF real, 10 PFC sham, 9 M1	MS diagnosis (121), with FSS > 4 or BDI-IA > 12 PFC real: - age: 47 (32–51) - EDSS: 2.5 (2.0–3.0) PFC sham: - age: 41 (39–45) - EDSS: 3.0 (2.5–3.0) M1 real: - age: 46 (42–48) - EDSS: 2.5 (2.5–3.5)	None serious: headache (30%), paresthesia or pain, gait disturbance, dizziness, tiredness, legs/bladder spasticity, discomfort
Korzhova et al. (118)	Bilateral M1	a) 20 Hz rTMS f8 coil b) iTBS + physical therapy (45–55 min/session)	1/day for 5 consecutive days, for 2 weeks a) 2 s on, 28 s off, 1,600 stimuli, 80% RMT, 30 min b) 10 bursts of 3 stimuli at 35 Hz, ITI 5 Hz, 1,200 stimuli/session, 80% RMT, 10 min	Yes (20 Hz rTMS group only)	MFIS Secondary outcome	Randomized, sham-controlled	34 SP (12 in the 20 Hz-rTMS group, 12 in the iTBS group, 10 in the sham group)	SP MS diagnosis according to McDonald criteria 2010 and lower spastic paraparesis with MAS > 2 measured in the knee - age: 45 (mean) - EDSS: 6.5 (mean)	None reported
**3) tRNS**									
Palm et al. (113)	F3 (EEG 10–20 system) 25 cm^2^	AF8 (EEG 10–20 system) 25 cm^2^	20 min/day for 3 days Peak to peak amplitude of 2 mA, full-band white noise from 0 to 500 Hz, variance 650/2 μA	No	MFIS	Crossover, sham-controlled (3-weeks wash-out)	16 (11 RR, 4 SP, 1 PP)	Clinically definite MS (121) and history of neuropathic pain with VAS > 40 age: 47.4 ± 8.9 EDSS: 4.2 ± 1.3 MFIS: 52.6 ± 12.3	Phosphenes, insomnia, nausea, severe headache (1, after sham)

*DLPFC, dorsolateral prefrontal cortex; EEG, electroencephalography; FSS, Fatigue Severity Scale; iTBS, intermittent theta burst stimulation; ITI, inter-train interval; MFIS, Modified Fatigue Impact Scale; MSFSS, MS-specific FSS; MRI, magnetic resonance imaging; PFC, prefrontal cortex; PP, primary progressive; PPC, posterior parietal cortex; PROMIS, Patient-Reported Outcomes Measurement Information System; RMT, resting motor threshold; RR, relapsing–remitting; RT,reaction time; SP, secondary progressive; rTMS, repetitive transcranial magnetic stimulation; tDCS, transcranial direct current stimulation; tRNS, transcranial random noise stimulation; VAS, visual analog scale for fatigue*.

The studies published so far are greatly heterogeneous, differing in many variables such as the NIBS technique used, the cortical targets, the stimulation intensity, and the characteristics of the populations included. Indeed although most of the studies enrolled patients with EDSS ≤ 6, other population characteristics were more heterogeneous among studies, such as the MS subtype, the presence of comorbidities, the measured outcome in addition to spasticity (e.g., neuropathic pain), and the baseline fatigue scores.

Other important limitations to the use of NIBS for therapeutic purpose remain the still heterogeneous definition of fatigue, the limited comprehension of its complex and multifactorial pathophysiology, and the limited use of objective measures other than self-report questionnaires.

Because of this methodological heterogeneity and the low sample sizes, the level of evidence for NIBS efficacy resulted too low to draw any robust conclusion to support its use in clinical practice (15) but encourages further studies on NIBS as a treatment for fatigue (120).

## Conclusions

Several studies have used neurophysiological tools to evaluate MS-related fatigue. Until now, this possibility has been mainly exploited for investigating the pathogenic mechanisms of fatigue and for modulating brain circuits for therapeutic purposes. The potential role of neurophysiology for quantifying fatigue and predicting and/or monitoring response to treatment has been evaluated in only a few studies.

From a methodological perspective, the most used techniques are TMS and tDCS. TMS is a very versatile method that allows both to assess, non-invasively, the functionality of corticospinal tract and cortico-cortical connections and, when delivered in repetitive protocols, to modulate brain activity (114). On the other side, tDCS is the most investigated technique as a potential treatment for fatigue because it is safe, well-tolerated, low-cost, and portable (13, 15).

Other neurophysiological techniques have been used, although in a relatively small number of studies. In particular, EEG has been used for exploring the role of cortico-cortical connections (34), EMG for evaluating the contribution of peripheral structures (9), evoked potentials for investigating the pathogenetic mechanisms (60) and predicting response to pharmacological treatment (94) and autonomic nervous system testing and polysomnography for assessing additional factors that can produce or exacerbate fatigue in PwMS.

Most part of the studies have been conducted in small samples by comparing the findings obtained in fatigued MS patients with those obtained in healthy controls or not-fatigued MS patients. Usually, each study used a single neurophysiological technique, while few studies combined different neurophysiological techniques (83) or neurophysiology with MRI (58).

Overall the literature data presented in this review demonstrate that neurophysiology could play a role in the management and evaluation of MS-related fatigue. Despite of heterogeneity in results and methodological limitations, current evidence supports further studies on the role of neurophysiology in the management of fatigue. In particular, for therapeutic purpose, tailored approaches based on individual network dysfunctions, individual plasticity impairment, and other neurophysiological variables should be explored.

## Author Contributions

FC, FM, EF, and MR wrote the manuscript. FC, FM, and VD critically revised the manuscript.

## Conflict of Interest

FC has received travel grants from Biogen, Merck, and Sanofi-Genzyme. The remaining authors declare that the research was conducted in the absence of any commercial or financial relationships that could be construed as a potential conflict of interest.
